# Selective nano-buckling improves the performance of graphene logic transistors

**DOI:** 10.1093/nsr/nwad316

**Published:** 2024-01-03

**Authors:** Tong Yang, Ming Yang

**Affiliations:** Department of Applied Physics, The Hong Kong Polytechnic University, China; Department of Applied Physics, The Hong Kong Polytechnic University, China; Research Centre for Nanoscience and Nanotechnology, The Hong Kong Polytechnic University, China

The massive integration of silicon-based transistors into modern electronic devices greatly increases power consumption and heat generation/dissipation [1]. In this concern, transistors based on atomically-thin graphene that exhibit high electric and thermoelectric conductivities with negligible contact resistance, wide-range gate-tunability and high compatibility with existing processing technologies, have emerged as promising alternatives in the post-Moore era [2,3]. Due to its gapless band dispersion, however, graphene-based transistors show a limited on/off switching ratio (<100). Various methods such as doping [4], substrate engineering [5] and dimensional reduction [6] have been proposed, but almost none of them could reach a satisfying on/off ratio without sacrificing carrier mobility. Further graphene functionalization, dedicated device architectures and even external field regulation are still required.

**Figure 1.**
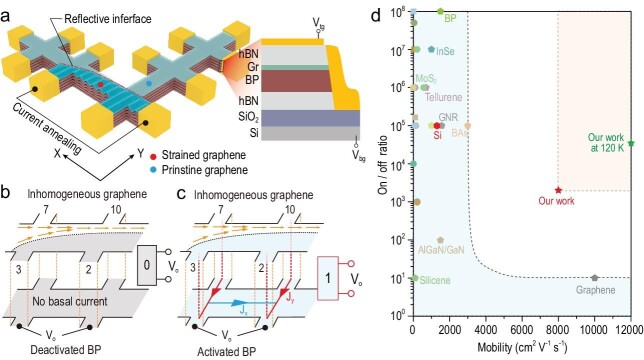
An illustration of the partially nano-buckled graphene transistor. (a) Schematic diagram of the graphene transistor. (b) Operation mechanism of graphene-based transistor. Inhomogeneous current flow within the graphene (Gr) channel when BP is insulating, representing Boolean ‘0’ state. (c) Dual-channel propagations of graphene and BP bottom layer. Non-zero *V*_o_ represents Boolean ‘1’ state. (d) Room-temperature on/off ratio and carrier mobility of FETs based on various semiconductors including metal oxides, transition metal dichalcogenides, and puckered semiconductors. Adapted from ref. [7].

Writing in *National Science Review*, a team led by Prof. Yanpeng Liu from Nanjing University of Aeronautics and Astronautics innovatively overcame this compromise between the on/off ratio and carrier mobility (Fig. 1). They proposed a novel device architecture based on a graphene/black phosphorus (BP) heterostructure that is peculiarly prone to nano-buckles on demand under local current-annealing. The as-fabricated graphene-based transistors show outstanding on/off ratios >10^3^ at room temperature while preserving intrinsic ultrahigh carrier mobility.

In the frame of electro-thermo-mechanical multiple fields coupling at the atomic scale, Prof. Liu and his coworkers first embarked on heavily lattice-mismatched heterostructures, for instance, a hexagonal graphene monolayer on orthogonal BP flakes, and selectively applied a direct current to thermally anneal graphene on puckered BP in between two electrodes. Upon global annealing, graphene/BP is likely to form a rotation-angle–dependent Moiré superlattice and pseudomagnetic field, accompanied with electron localization within circumscribed graphene landscapes [8]. Here, the annealed graphene/BP region locally buckles at the nanoscale with a ridge-like spatial structure (behaving as electron reflective interface) formed at the intersection with the adjacent un-annealed one. This electrical interface blocks the in-plane current flow and gives rise to an off-state in the logic transistors. For the on-state, the authors alternatively leverage a simple back-gate to make the BP bottom layer conductive thus generating dual-channel propagations of graphene and BP. In this manner, the logic operation of the graphene transistor is readily realized by low-power gate control, even at room temperature and intense magnetic fields.

Via electrostatic gating, the graphene-based transistor simultaneously generates an on/off ratio >10^3^ and a high mobility of ∼8000 cm^2^ V^−1^ s^−1^ at room temperature, fulfilling the low-power criterion suggested by the International Roadmap for Devices and Systems [9]. This breakthrough not only sheds light on the control of two-dimensional electrons through nanoscale multiple-fields coupling, but also establishes a paradigm for the development of graphene-based transistors, edging us one step closer to their ultimate commercialization.
